# Triangulating patient and public involvement in clinical research: a cross-cohort qualitative study

**DOI:** 10.1186/s40900-026-00918-0

**Published:** 2026-06-03

**Authors:** Ben Hood, Andy Bojke, Catherine Cooper, Lesley Curtis, William Cruickshank, Stephen Searl, Sue Searl, Rona Bojke, Irene Soulsby, Chris Walker, Jaime Amezaga

**Affiliations:** 1 Sir Bobby Robson Cancer Trials Research Centre, Northern Centre for Cancer Care, Freeman Hospital, Newcastle upon Tyne, UK; 2 Patient and Public Contributors, Newcastle upon Tyne, UK

## Abstract

**Background:**

Patient and Public Involvement (PPI) is widely endorsed within UK clinical research as both an ethical expectation and a mechanism for enhancing research relevance and quality. However, questions remain regarding how involvement functions in practice across institutional settings. Most studies examine PPI from single stakeholder perspectives, limiting understanding of how involvement is experienced across research systems. The DANIELLE study was co-produced with an established cancer PPI group and aimed to triangulate perspectives from multiple actors to examine how PPI is valued, evidenced and sustained within clinical research governance.

**Methods:**

A qualitative multi-cohort design was used. Forty semi-structured interviews were conducted with purposively sampled participants, including lay PPI contributors, clinical trial sponsor staff, professional PPI leads and Research Ethics Committee members. Participants were recruited through established PPI networks, NHS research governance contacts and professional research channels. Data were analysed using reflexive thematic analysis, with cross-cohort triangulation employed to identify shared structural patterns. Reflexive and co-production principles informed study design and interpretation.

**Results:**

Five interconnected themes were generated: (1) PPI as ethically essential but structurally fragile; (2) authenticity versus performative compliance; (3) evidentiary gaps and the invisibility of contributor labour; (4) timing of involvement as a marker of authenticity; and (5) reliance on individual goodwill rather than institutional infrastructure. Across cohorts, participants described strong ethical commitment to involvement alongside systemic weaknesses in how experiential contributions are documented, recognised and sustained.

**Conclusion:**

The findings suggest that persistent challenges in PPI reflect features of research system design rather than isolated failures of engagement. Strengthening involvement requires movement from rhetorical endorsement toward infrastructural integration, including improved approaches to evidencing influence and earlier positioning of contributors within research development pathways. By triangulating stakeholder perspectives through a co-produced inquiry, the study conceptualises PPI as a shared system-level knowledge practice rather than an optional enhancement to research delivery.

**Supplementary Information:**

The online version contains supplementary material available at 10.1186/s40900-026-00918-0.

## Abstract (plain language summary)

Patient and Public Involvement (PPI) means including patients and members of the public in shaping health research. In the UK, PPI is considered an ethical expectation, but there is ongoing debate about how well it works in practice. This study explored how PPI is experienced across the clinical research system. We interviewed four groups involved in research: public contributors, NHS research staff, professional PPI leads, and Research Ethics Committee members. By comparing their perspectives, we examined how involvement is valued, recorded, and supported. All groups agreed that PPI is important and can improve research. However, participants described weaknesses in how involvement is protected within institutions. Public contributors often felt their work became invisible once studies entered formal approval systems. Professionals described a lack of clear ways to show how PPI changes research. Ethics committees reported difficulty checking whether involvement was meaningful or simply a requirement to complete. Participants also emphasised that early involvement leads to stronger research, while late consultation risks becoming a “tick-box” exercise. Across the system, meaningful PPI was often sustained by individual goodwill rather than strong institutional structures. The findings suggest that improving PPI requires system-level change. Better ways of documenting impact, involving contributors earlier, and recognising contributor labour are needed. PPI should be supported as a core part of research infrastructure, not an optional extra.

**Supplementary Information:**

The online version contains supplementary material available at 10.1186/s40900-026-00918-0.

## Introduction

### Patient and public involvement in clinical research systems

Patient and Public Involvement (PPI) is widely recognised as a core principle within contemporary clinical research governance. PPI refers to the active involvement of patients and members of the public in shaping research, rather than their participation as study subjects. Within UK health research policy, involvement is framed both as an ethical expectation and as a mechanism for improving research relevance, quality and accountability to the populations it seeks to serve [1–4]. In this context, involvement may be understood as operating at two interconnected levels. First, it reflects a democratic or rights-based rationale, whereby individuals affected by research are afforded opportunities to influence decisions shaping healthcare services and future treatment pathways. Second, involvement has epistemic value, contributing experiential knowledge that can enhance the feasibility, acceptability and usefulness of research interventions and processes [5].

Despite broad policy endorsement, evidence suggests that the implementation of PPI remains variable across institutional settings. Previous research has highlighted ongoing tensions between expectations for meaningful collaboration and organisational pressures associated with funding timelines, governance requirements and research delivery targets [1, 6, 7]. Much of the existing literature has focused on single stakeholder perspectives, often privileging either contributor experiences or professional governance viewpoints. Consequently, involvement is frequently examined as an interpersonal process rather than as a phenomenon shaped by wider research system structures, including regulatory frameworks, evidencing norms and institutional cultures [8, 9].

A growing body of research has also drawn attention to challenges associated with evidencing the influence of PPI. Involvement is commonly described through narrative accounts or reflective reporting rather than through consistent or traceable documentation, creating uncertainty for oversight bodies seeking to understand how experiential input has shaped research design and decision-making [4, 10]. These tensions reflect broader differences in how evidence is conceptualised within research systems, where procedural or measurable indicators may be prioritised over contextual and relational forms of knowledge. Research Ethics Committees (RECs), for example, are required to assess ethical acceptability while often relying on researcher-reported descriptions of involvement activities presented within application processes [7]. Recent international mapping work has highlighted the continued expansion of PPI policy alongside persistent uncertainty regarding its operationalisation and evaluation [11]. Emerging research examining funder governance contexts further suggests that institutional expectations for involvement are shaped by national policy infrastructures and organisational cultures [12].

Timing of involvement has also been identified as a critical determinant of influence. Early engagement is associated with greater capacity to shape research questions, study design and participant experience, whereas later consultation may limit the scope for substantive change and contribute to perceptions of symbolic or performative involvement [1, 8]. However, institutional systems do not consistently require detailed reporting of when and how involvement occurred, nor how contributor insights informed research development. As a result, PPI may be strongly endorsed at policy level while remaining operationally uneven within research pathways [10].

Understanding involvement therefore requires attention to the wider research ecosystem in which it is enacted. Clinical research is shaped by a network of stakeholders — including contributors, researchers, sponsors, funders and governance bodies — each occupying distinct structural positions that influence how PPI is interpreted and operationalised [4, 7]. Recent multi-stakeholder studies highlight the value of examining how expectations, incentives and interpretations of involvement circulate across this system, revealing tensions that may remain obscured when perspectives are studied in isolation [13–16]. However, there remains limited empirical work examining how these perspectives intersect across stakeholder groups within a single study, particularly in relation to how involvement is evidenced, interpreted and sustained within research governance systems.

The DANIELLE study emerged from sustained reflection within an established cancer PPI group with more than a decade of experience reviewing clinical research. Contributors identified concerns relating to perceived tokenism, limited transparency regarding how experiential input influenced study design, and the structural invisibility of contributor labour within governance processes. Named in memory of a long-standing contributor whose advocacy shaped the research direction, the study reflects a co-produced inquiry grounded in contributor-identified priorities and relational engagement.

The objective of this paper is to examine how Patient and Public Involvement is understood, valued and evidenced across multiple stakeholder groups within UK clinical research governance, and to identify system-level factors shaping its implementation and sustainability.

This study aimed to explore how PPI is understood, valued and evidenced across key stakeholder groups within UK clinical research governance systems. By triangulating perspectives from contributors, sponsorship staff, professional PPI leads and REC members, the study examined involvement as a system-level phenomenon and sought to identify structural factors influencing its implementation and sustainability.

### The study addressed the following research questions

- How is PPI interpreted across stakeholder groups within clinical research governance?
- How is involvement evidenced, evaluated and legitimised within institutional processes?
- What structural factors shape the sustainability and perceived authenticity of PPI?

## Methods

### Study design

The DANIELLE study is a multi-cohort qualitative investigation examining how PPI is understood, valued and operationalised within UK clinical research systems. The study was conducted between 2024 and 2025 within UK clinical research governance settings. The study was designed to explore not only procedural experiences of involvement but also how different forms of knowledge, including experiential, professional and regulatory perspectives, shape interpretations of PPI within research governance. Semi-structured interviews and reflexive thematic analysis were used to explore experiences of PPI across multiple stakeholder groups involved in the design, governance and oversight of clinical research.

A triangulated qualitative design was adopted. Triangulation is recognised as an established qualitative strategy for enhancing depth of understanding through the integration of multiple data sources and perspectives [17, 18]. In this study, triangulation enabled examination of how PPI was interpreted across structurally distinct positions within the clinical research system. In this context, triangulation refers to the integration of themes derived from multiple stakeholder perspectives to develop a richer understanding of PPI as a system-level phenomenon. This approach enabled examination of how involvement is constructed, legitimised and evidenced across institutional contexts rather than within isolated interactions.

Rather than privileging a single viewpoint, the study sought to examine how PPI is experienced across different positions within the research ecosystem and how these perspectives converge, diverge or reveal structural tensions. The study was conducted and reported in accordance with the Consolidated Criteria for Reporting Qualitative Research (COREQ) guidance.

### Patient and public involvement in study design

This study was co-produced with members of an established cancer PPI group. Contributors were involved in shaping the research aims, refining interview schedules, and advising on interpretation of findings. Their involvement was grounded in relevant lived experience of engaging with clinical research processes, enabling the study to draw on experiential knowledge to inform both conceptual framing and methodological decisions. The research question emerged directly from collective reflection within the group regarding their experiences of interacting with clinical research governance systems.

Contributors identified the need to examine how PPI is operationalised, valued and evidenced across institutional contexts. Involvement extended beyond consultation to active collaboration throughout the study lifecycle. Contributors informed decisions regarding stakeholder sampling, the framing of questions relating to ethical acceptability and evidencing of impact, and the interpretation of system-level themes emerging from the data. Throughout the study, ongoing dialogue with PPI members informed analytic interpretation and supported reflexive consideration of how researcher assumptions and institutional norms might shape meaning-making.

The project therefore represents a co-produced inquiry into systems that directly affect PPI contributors themselves. Reporting of PPI follows GRIPP2 guidance.

### Participants and sampling

Participants were purposively sampled to capture perspectives from stakeholders occupying distinct roles within the clinical research infrastructure. Purposive sampling enables the selection of information-rich participants with direct experience of the phenomenon under investigation [19].

### Four cohorts were included

1. Lay members from a cancer PPI group.
2. NHS staff involved in managing clinical trial sponsorship.
3. Professional PPI leads.
4. REC members.

Each cohort represented a distinct position within the research pathway, enabling examination of how PPI is interpreted, enacted and evaluated across multiple structural levels.

Ten participants were recruited from each cohort (*n* = 40), providing sufficient depth within cohorts while enabling cross-cohort comparison. Participants were recruited via PPI networks, NHS research governance contacts and professional channels. Inclusion criteria required demonstrable experience engaging with, managing or reviewing PPI within clinical research contexts.

### Data collection

Data were collected through semi-structured interviews conducted between 2024 and 2025. Interview schedules were tailored to each cohort but maintained conceptual alignment to support triangulation. The interview guides were developed collaboratively with PPI contributors and piloted with two members of the contributing group to ensure clarity, relevance and sensitivity of wording.

The final topic guide is provided in Appendix 1. Questions explored:

- Experiences of engaging with PPI.
- Perceived value and legitimacy of PPI.
- Structural facilitators and barriers.
- How PPI is evidenced and interpreted within research systems.
- Recommendations for improvement.

Interviews lasted approximately 30–60 min and were conducted either face-to-face or via secure video conferencing. All interviews were audio-recorded with consent and transcribed verbatim. Transcripts were anonymised using coded identifiers.

The semi-structured format enabled participants to articulate experiences in their own terms while ensuring consistency of thematic coverage across cohorts.

### Data analysis

Data were analysed using reflexive thematic analysis following Braun and Clarke’s six-phase framework [20]:

1. Familiarisation with the data.
2. Initial coding.
3. Theme development.
4. Theme review.
5. Theme definition and naming.
6. Reporting.

Analysis was conducted iteratively, with movement back and forth between phases rather than as a strictly linear process, consistent with reflexive thematic analysis. To support the triangulated design of the study, analysis proceeded at two interconnected levels rather than as discrete sequential stages.

First, within-cohort analysis was undertaken, in which each stakeholder group was analysed independently to identify patterns of meaning reflecting internal experiences and interpretive frames. Second, cross-cohort triangulation was conducted, whereby themes generated within each cohort were compared to identify convergences, tensions and structural contradictions across the research system.

These analytic levels were embedded within Braun and Clarke’s six-phase framework and represent a strategy for integrating multiple perspectives, rather than a separate analytic method. Themes generated within cohorts were subsequently compared to identify convergences, tensions and structural contradictions across the research system. This triangulated approach enabled examination of how PPI is constructed, evaluated and operationalised across institutional contexts rather than understood solely as an individual practice. Theme development was discussed with qualitative advisors to enhance reflexive rigour. Disagreements were explored through analytic dialogue rather than consensus coding, consistent with reflexive thematic analysis principles.

**Fig. 1:**
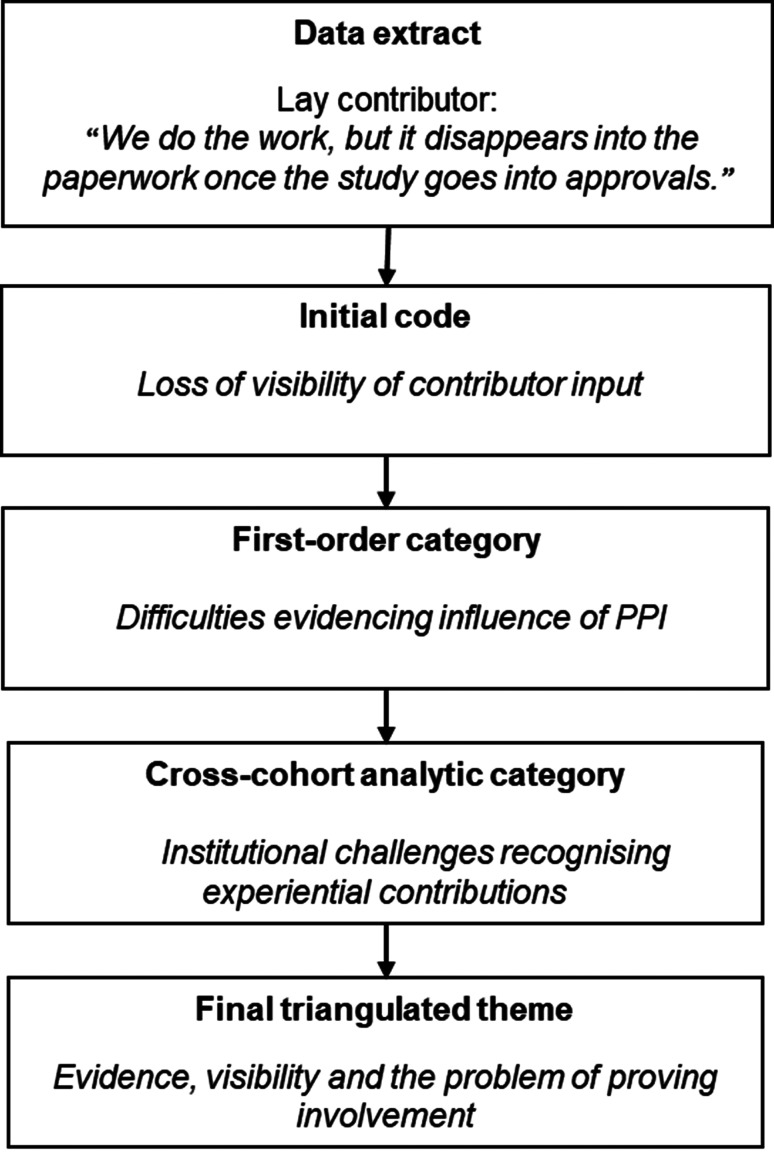
Illustrative example of thematic development in the DANIELLE study

An illustrative example of this analytic progression is presented in Fig. 1, demonstrating how inductive codes derived from interview data were iteratively developed into higher-order analytic categories and integrated through cross-cohort comparison to generate final system-level themes.

### Rigour and reflexivity

Qualitative rigour was supported through transparent documentation of analytic decisions, sustained reflexive engagement with the data and systematic recording of coding development. The lead researcher is a senior clinical research nurse with extensive professional involvement in cancer PPI and research governance activity. Reflexive journaling was used throughout the study to critically examine how researcher positionality, professional proximity and prior knowledge might shape interpretation.

In line with reflexive thematic analysis principles, subjectivity was approached as an analytic resource that enabled deeper engagement with experiential accounts rather than as a source of bias to be eliminated.

Triangulation across cohorts strengthened analytic credibility by enabling patterns of meaning to be explored from multiple institutional vantage points. Convergences and tensions identified across stakeholder groups were treated as analytically significant indicators of system-level dynamics. An audit trail of analytic memos and theme development was maintained within NVivo to support transparency of interpretation.

The researcher’s long-standing professional relationship with the contributing PPI group created both analytic proximity and ethical accountability. Reflexive practice was therefore used to consider how insider positioning informed meaning-making and interpretive focus [21, 22]. Naming the study after Danielle acknowledges the relational foundations of the research and situates the inquiry within an ongoing collaborative partnership with contributors [23].

### Ethical considerations

Ethical approval for the DANIELLE study was granted by the Health Research Authority (HRA) via the Integrated Research Application System (IRAS) (Project ID: 335889). All participants provided informed consent prior to interview.

Participants were informed of their right to withdraw without consequence. Anonymity was preserved through coded identifiers and removal of identifying details. Data were stored securely in accordance with NHS research governance requirements. Ethical conduct of the study was further supported through ongoing dialogue with contributor partners, recognising the relational and experiential dimensions of participation within co-produced research.

### Findings

**Fig. 2:**
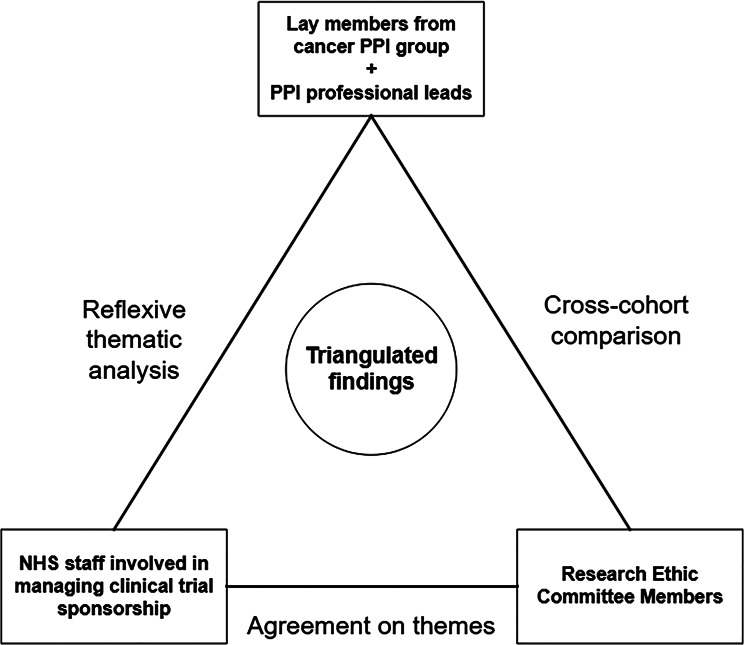
Triangulated thematic model showing how PPI is experienced across stakeholder groups in clinical research

The findings are organised around five triangulated themes generated through cross-cohort thematic analysis. These themes represent shared system-level patterns visible across stakeholder groups rather than isolated experiences. They reflect how involvement is interpreted, legitimised and experienced within institutional contexts shaped by differing forms of knowledge and authority. Figure 2 presents the triangulated thematic model, illustrating how perspectives from contributors, sponsors, PPI leads and REC members converge to produce integrated system-level interpretations. Table 1 provides the cross-cohort evidence matrix underpinning each theme. Triangulation was used to explore relational and structural dynamics within the research system rather than to seek a single objective or standardised account of involvement. Taken together, the themes illustrate how involvement moves through the research system from ethical expectation to practical implementation and how this movement shapes stakeholder experiences.

**Table 1:** Cross-cohort triangulation of PPI experiences in the DANIELLE study

Thematic focus	Cancer PPI Lay Members	NHS Sponsor Staff	Professional PPI leads	Research Ethics Committee members	Integrated triangulated interpretation
Authenticity versus performative compliance	Describe both meaningful influence and token consultation	Observe variability in researcher commitment to involvement	Work to challenge symbolic or compliance-driven approaches	Report difficulty verifying authenticity through application documentation	Research systems may incentivise procedural demonstration of involvement rather than relational influence on research decision-making
Ethical meaning of PPI	View involvement as protective of participants and morally important	Recognise ethical value but describe inconsistent implementation	Advocate strongly for the ethical centrality of PPI	Often treat PPI as a proxy indicator of participant acceptability in review	PPI holds shared democratic and experiential ethical value across cohorts but remains unevenly embedded within organisational structures
Evidence and visibility	Experience contributor input becoming obscured within governance processes	Lack consistent mechanisms for demonstrating impact	Report institutional difficulty evidencing involvement outcomes	Forced to rely on researcher self-report	Privileging of measurable evidence limits recognition of narrative and experiential contributions
Timing of involvement	Early engagement associated with genuine influence	Late consultation linked to operational and funding pressures	Promote early integration but lack authority to require it	Identify technical language in applications as signalling late involvement	Early involvement operates as a system-level indicator of partnership and epistemic influence
Responsibility for sustaining PPI	Reliant on volunteer goodwill	Involvement dependent on motivated individuals within teams	Provide leadership but often lack structural power	Limited capacity to enforce meaningful involvement	Meaningful involvement is frequently sustained through individual advocacy rather than formal organisational commitment

### Triangulated system-level themes

The five themes presented below represent interconnected system-level patterns identified through cross-cohort analysis rather than discrete or isolated categories of experience. Each theme integrates perspectives from contributors, sponsor staff, professional PPI leads and REC members to illustrate how involvement is interpreted and operationalised across different positions within the clinical research system.

The themes are organised to guide the reader from foundational ethical understandings of involvement (Theme 1), through tensions in how involvement is enacted in practice (Theme 2), to challenges associated with evidencing influence within governance processes (Theme 3). Themes 4 and 5 then extend this analysis by examining the structural significance of when involvement occurs and who is understood to hold responsibility for sustaining meaningful engagement.

Taken together, this progression reflects how PPI moves through research systems — from normative expectation to practical implementation and how institutional contexts shape both its visibility and perceived legitimacy.

#### Theme 1: *PPI as ethically essential but structurally fragile*

Across all stakeholder groups, PPI consistently constructed as an ethically fundamental component of clinical research, shaping how participants understood both the purpose and legitimacy of involvement within research systems. Lay contributors framed their participation as grounded in lived experience and a sense of responsibility to improve future care. Involvement was frequently understood as a form of moral reciprocity following personal encounters with cancer services. As one contributor reflected, “It’s giving something back. We’ve been through it — if we don’t speak up, who will?”

Participants described their role not only as advisory but also as protective, emphasising the importance of safeguarding dignity and humanity within research processes. In this sense, involvement was positioned as a means of ensuring that experiential perspectives remained visible within technically and procedurally complex research environments. This was captured in the view that PPI existed to ensure researchers did not “forget the patient in all the science.”

Professional stakeholders broadly shared this ethical framing. Sponsor staff and PPI leads characterised involvement as a corrective to insular research cultures, highlighting the value of experiential knowledge in shaping study feasibility and participant acceptability. For these participants, PPI functioned both as an ethical commitment and as a practical resource for improving research design. Within governance contexts, REC members frequently described the presence of PPI as signalling ethical awareness within applications. However, this endorsement was often pragmatic rather than evidential, with REC participants noting that involvement functioned as an indirect indicator of ethical intent rather than as demonstrable proof of influence on study design.

Despite this shared commitment, participants across cohorts also described involvement as structurally vulnerable. Lay contributors spoke of the emotional significance of their engagement alongside uncertainty regarding how their input translated into institutional decision-making. Several described a sense of disappearance once projects progressed into formal governance processes. Similarly, PPI leads highlighted constrained authority within organisational hierarchies, noting that while involvement was rhetorically prioritised, mechanisms to require or verify meaningful engagement remained limited. As one lead observed, “We’re told it’s essential, but structurally it still feels optional.”

Taken together, these accounts reveal a consistent system-level pattern. Although PPI was widely positioned as ethically non-negotiable, participants simultaneously described organisational arrangements that did not consistently recognise, evidence or sustain contributor influence. The theme therefore reflects a central tension within contemporary clinical research systems: involvement is collectively endorsed as morally important yet remains unevenly embedded within the institutional structures responsible for supporting its implementation.

#### Theme 2: *Authenticity versus performative compliance*

Extending the structural fragility identified in Theme 1, participants described involvement in practice as frequently shifting between authentic collaboration and procedural performance. Across all stakeholder groups, PPI was understood as existing on a continuum between genuine partnership and symbolic inclusion. Rather than presenting PPI as simply effective or ineffective, participants emphasised how its quality was shaped by researcher behaviours, organisational expectations and the timing of engagement within the research lifecycle.

Lay contributors provided vivid contrasts between experiences that fostered collaboration and those perceived as primarily procedural. Meaningful involvement was associated with early dialogue, openness to challenge and demonstrable modification of research plans. Participants described feeling respected when researchers made visible how experiential insights had informed study decisions. As one contributor explained, “The good ones come back and show you what they changed. You can see your voice in the study.” Such encounters were characterised as reciprocal and relational, reinforcing a sense of shared ownership and epistemic contribution. In these contexts, contributors reported feeling recognised as partners rather than symbolic consultees, reflected in the observation that “when they actually listen, you feel like a partner, not a prop.”

In contrast, participants also described encounters marked by limited preparation, defensiveness or only superficial engagement with feedback. These experiences were commonly interpreted as evidence that involvement had been undertaken primarily to satisfy procedural requirements. Contributors noted that symbolic consultation could often be inferred through subtle interactional cues, including researchers appearing disengaged or reluctant to consider suggested changes. More negatively experienced encounters were described as dismissive or patronising, with participants suggesting that such interactions could undermine confidence and reduce willingness to contribute to future research activities.

Professional stakeholders similarly acknowledged substantial variability in how involvement was conceptualised and enacted across research teams. Sponsor staff described differing levels of researcher commitment, noting that organisational pressures — including funding timelines and governance milestones — could shift involvement towards compliance-oriented activity rather than collaborative learning. REC members reported difficulty in distinguishing authentic engagement from narrative descriptions of involvement within applications. In the absence of structured evidencing mechanisms, reviewers described relying on interpretive judgement and contextual cues when assessing whether reported involvement reflected substantive partnership or administrative performance.

Taken together, these accounts illustrate a persistent tension between involvement as a relational practice grounded in dialogue and mutual learning, and involvement as an institutional requirement embedded within research processes. This tension was recognised across stakeholder groups, although experienced differently depending on organisational position and responsibility within the research system. The theme therefore highlights how systemic expectations and operational pressures shape both the quality and perceived legitimacy of involvement within contemporary clinical research settings.

#### Theme 3: *Evidence*,* visibility and the problem of proving involvement*

In addition to variability in the quality of involvement described in Theme 2, participants across stakeholder groups raised concerns about how the influence of PPI could be recognised, demonstrated and legitimised within research governance systems. A dominant cross-cohort concern related to the apparent disconnect between relational engagement as it was experienced in practice and the forms of documentation used to represent involvement within institutional processes. Although involvement activity was widely described as occurring, its consequences were not always visible, traceable or formally acknowledged within approval pathways.

Lay contributors frequently articulated uncertainty about whether their feedback resulted in tangible modifications to study design. Several described a sense that their contributions became obscured once projects progressed into governance procedures. One participant reflected that “we do the work, but it disappears into the paperwork once they got what they needed from us,” highlighting the perceived loss of visibility as research moved through organisational structures. This uncertainty was rarely framed as mistrust of individual researchers; rather, it was interpreted as a structural limitation in how experiential insights were recorded, communicated and valued within institutional systems.

Professional stakeholders similarly emphasised ongoing challenges in demonstrating the influence of involvement. Sponsor staff acknowledged the absence of consistent expectations or mechanisms for documenting change, noting that descriptions of PPI often remained narrative, context-dependent and unevenly reported. Participants suggested that this lack of standardisation generated ambiguity for governance reviewers attempting to assess the extent and significance of contributor impact. PPI leads described additional tensions arising from the inherently relational character of involvement work. Dialogue, negotiation and shared learning were frequently experienced as central to meaningful engagement, yet participants noted that such processes did not translate easily into structured reporting formats. As one lead observed, “the most meaningful conversations don’t fit neatly into forms.”

REC members reported particular discomfort with reliance on researcher self-report when evaluating involvement within applications. In the absence of verifiable indicators or transparent documentation pathways, reviewers described drawing on interpretive cues — including narrative framing, language use and perceived intent — when judging the authenticity of engagement. This evaluative uncertainty was recognised across cohorts as a systemic feature rather than an individual failing. Participants consistently depicted a research environment in which experiential knowledge was valued rhetorically but remained difficult to evidence in procedurally meaningful ways.

Collectively, these accounts illustrate how involvement may be experientially present yet administratively fragile. The theme therefore reflects broader epistemic tensions between relational, contextual forms of knowledge and institutional expectations for standardised, auditable documentation. As a consequence, uncertainty persists regarding how experiential contributions shape research design and how such influence can be credibly recognised within governance systems.

#### Theme 4: *Timing matters — early involvement as a marker of authenticity*

Participants further identified the timing of engagement as a key mechanism shaping whether involvement became meaningful influence or symbolic consultation.

Across all stakeholder groups, the timing of involvement emerged as a key interpretive signal of whether PPI was experienced as meaningful partnership or procedural requirement. Participants consistently associated early engagement in the research lifecycle with greater opportunity for influence, shared ownership and collaborative learning. In contrast, involvement introduced at later stages was often perceived as limited in scope and primarily confirmatory.

Lay contributors emphasised that the potential to shape research was closely tied to when they were invited to participate. Early dialogue was described as enabling co-creation of ideas and fostering a sense of relational investment in the study. Participants contrasted this with experiences in which protocols appeared largely finalised prior to consultation, positioning involvement as reactive rather than formative. As one contributor noted, “*If it’s already written*,* what are we actually changing?”* In such contexts, involvement was experienced as corrective rather than generative, with limited opportunity to influence fundamental aspects of study design.

Professional stakeholders similarly recognised the importance of early engagement while acknowledging structural constraints. Sponsor staff described how operational pressures, including funding deadlines and governance milestones, could unintentionally result in involvement occurring later than intended. Although not typically framed as deliberate exclusion, these systemic factors were understood to reduce the extent to which experiential knowledge could shape research decisions. PPI leads reported ongoing efforts to encourage research teams to involve contributors at the conceptual stage rather than during final protocol refinement.

REC members also identified timing as an indirect indicator of engagement quality. Participants described interpreting aspects of application language and study development narratives as signals of whether involvement had occurred early enough to inform design. Across cohorts, early involvement was therefore widely understood as evidence of authentic partnership, while delayed engagement was more likely to be interpreted as compliance with procedural expectations.

Taken together, these accounts highlight timing as a structural feature shaping how involvement is experienced and evaluated within clinical research systems. The theme illustrates how institutional timelines and development pathways can influence not only the practical scope of contributor influence but also perceptions of the legitimacy and authenticity of involvement.

#### Theme 5: *Institutional responsibility versus individual goodwill*

These tensions were compounded by perceptions that responsibility for sustaining involvement rested more with individuals than with organisational systems.

Across stakeholder groups, participants described PPI as frequently sustained through personal commitment rather than consistently embedded organisational structures. Contributors often framed their engagement as motivated by responsibility to future patients and a desire to improve the research system. This sense of purpose was described as emotionally meaningful but also fragile, particularly in the absence of visible institutional recognition. As one lay participant reflected, *“We’re doing this because we care — but caring shouldn’t be the system*.”

Professional stakeholders similarly characterised involvement as dependent on individual advocacy and leadership. PPI leads described investing significant effort in encouraging research teams to engage contributors early and meaningfully, often relying on persuasion rather than formal authority. Sponsor staff acknowledged that while involvement was widely supported in principle, competing operational priorities including timelines, funding pressures and service delivery targets — could limit the extent to which it was prioritised in practice. In this context, meaningful engagement was often attributed to motivated individuals rather than to organisational mandate or protected resources.

REC members also recognised limitations in the extent to which governance systems could enforce expectations for involvement. While reviewers reported encouraging strong PPI, they noted a lack of structural mechanisms to require consistent implementation. Across cohorts, participants therefore depicted a research environment in which involvement persisted through goodwill, professional values and informal leadership rather than through clearly defined institutional responsibility.

Taken together, these accounts highlight the vulnerability of relying on personal commitment to sustain involvement within complex research systems. Participants suggested that strengthening PPI would require greater organisational recognition, resourcing and integration into routine governance processes. The theme underscores a broader shift from viewing involvement as an optional enhancement to understanding it as an infrastructural component of research design and delivery.

## Discussion

### Overview of main findings

This study explored how PPI is understood, experienced and operationalised across multiple stakeholder groups within UK clinical research governance. Across five triangulated themes, participants described involvement as widely endorsed in principle yet unevenly embedded in institutional practice. PPI was positioned as ethically essential and experientially meaningful, but structurally fragile. Stakeholders consistently identified tensions between meaningful collaboration and performative compliance, alongside persistent challenges in evidencing influence, integrating involvement early in research development, and sustaining engagement beyond individual goodwill. Participants also highlighted the influence of funding structures and timelines in shaping when and how involvement occurs, suggesting that funders represent an important yet under-examined system-level actor in the organisation of PPI practice.

Importantly, the findings indicate that the ethical value attributed to PPI operates at more than one level. Participants framed involvement both as a moral expectation — grounded in the rights of people affected by research to influence decisions — and as a practical mechanism for improving research relevance, feasibility and quality through the integration of experiential knowledge. These interpretations shaped how stakeholders understood the purpose of involvement and the consequences of its absence within research systems.

### Comparison with existing literature

The findings resonate with longstanding concerns in the PPI literature regarding tokenism, variability in engagement quality and the invisibility of contributor labour [1, 8]. More recent work continues to highlight these persistent challenges despite expanding policy emphasis on involvement [11, 14]. However, by triangulating perspectives from contributors, sponsors, professional PPI leads and REC members, the present study extends this body of work by providing an integrated, system-level account of how these challenges are experienced across institutional roles rather than within single stakeholder groups. This suggests that limitations in PPI practice may reflect system-level design features rather than isolated shortcomings within individual research teams.

The study also contributes to debates regarding the ethical function of involvement. While PPI is often treated within governance processes as a proxy indicator of participant acceptability, participants described broader contributions to ethically sensitive aspects of study design, including language, burden of participation and perceived fairness. This finding extends previous conceptual work by illustrating how experiential knowledge operates in practice within governance contexts, rather than remaining an abstract ethical principle. These findings support arguments that experiential knowledge can enhance ethical review by complementing formal regulatory expertise with insight grounded in lived experience.

Consistent with previous research, participants highlighted ongoing difficulties in evidencing the influence of PPI [4]. The absence of shared approaches to documenting change was experienced as problematic across stakeholder groups, particularly for ethics reviewers tasked with assessing engagement without direct access to involvement processes. Similar tensions have been identified in relation to the narrative and relational character of PPI evidence [10]. The present study builds on this literature by demonstrating how these evidentiary tensions are reproduced across multiple points within the research system, including sponsorship, governance and contributor perspectives. The present findings suggest that this evidentiary challenge may reflect deeper epistemological differences within research systems, where measurable indicators are prioritised over contextual or experiential forms of knowledge.

Timing of involvement also emerged as a key structural issue. In line with previous studies linking early engagement to improved research relevance and participant-centred design [1], stakeholders interpreted early collaboration as evidence of partnership and delayed consultation as indicative of procedural compliance. By examining this across stakeholder groups, the study highlights how timing functions not only as a practical constraint but also as a shared interpretive marker of authenticity within research systems. Institutional timelines that position PPI later in development may therefore inadvertently encourage performative models of involvement.

Finally, the findings highlight the continued reliance of PPI on individual advocacy and personal commitment, echoing wider critiques that involvement systems often lack durable organisational scaffolding [7]. Recent research examining organisational and funder contexts similarly identifies structural limitations in how involvement is embedded and sustained [12]. While goodwill enables engagement to persist, it may not ensure sustainability or consistency across research settings. The present study contributes to this discussion by showing how reliance on individual actors is recognised across stakeholder groups, reinforcing its character as a systemic rather than localised issue.

### Strengths and limitations

A key strength of this study is its triangulated qualitative design, which enabled examination of PPI across multiple stakeholder positions within the clinical research system. By integrating perspectives from contributors, governance professionals and institutional leads, the study provides a system-level account of involvement that extends beyond single-cohort analyses. The co-produced origins of the research further strengthen its relevance to lived experiences of involvement.

However, the study is situated within UK clinical research governance and may not be directly transferable to contexts with different regulatory infrastructures. Participants were purposively sampled to prioritise depth of insight rather than statistical representativeness, consistent with qualitative methodology. While the study was designed to explore involvement as a system-level phenomenon, the analysis necessarily reflects the conceptual framing adopted by the research team.

Ethical and structural dimensions of involvement were foregrounded, and alternative theoretical perspectives, for example those focusing more explicitly on methodological or epistemological functions of experiential knowledge may have generated different interpretive emphases.

As with all qualitative research, interpretation is shaped by researcher reflexivity. The lead researcher’s professional proximity to PPI practice provided contextual insight but may also have influenced analytic attention towards systemic tensions. Reflexive processes were used to critically examine these influences, although complete neutrality is neither assumed nor claimed within reflexive thematic analysis. Cross-cohort triangulation strengthens analytic credibility by demonstrating convergent patterns across stakeholder groups.

The study also focuses primarily on formal institutional settings and may not capture informal or community-led models of involvement operating outside governance frameworks. Perspectives from research funders, regulators beyond REC membership, and researchers with limited engagement in involvement were not included and may represent additional influences on how PPI is conceptualised and evidenced. Future research could examine how involvement is structured and sustained in alternative research environments and explore how different forms of evidence including narrative accounts of experiential influence are recognised and evaluated within research governance systems.

### Recommendations

The findings suggest that strengthening PPI requires institutional reform rather than additional advocacy alone. They also indicate a need to reconsider how involvement is conceptualised, evidenced and valued within research systems. Rather than introducing entirely new principles, these findings extend existing debates by highlighting how known challenges are reproduced across multiple stakeholder positions within research governance systems. Based on cross-cohort triangulation, the following system-level implications can be drawn:

1.*Evidencing involvement as contextual and narrative practice*

The findings suggest a need to move beyond standardised or purely quantitative approaches to evidencing PPI. Consistent with wider critiques of evidencing practices [10], participants across stakeholder groups described the importance of narrative and contextual accounts in demonstrating how experiential knowledge shapes research design. Developing flexible approaches to documenting influence may therefore better align reporting systems with the relational nature of involvement.

2.*Earlier integration of involvement within research pathways*

In line with existing literature linking early engagement to improved research relevance [1], the findings indicate that involvement is most influential when embedded at the conceptual stage of research development. Participants across cohorts identified late-stage consultation as limiting the scope for meaningful contribution, suggesting that institutional processes may benefit from stronger expectations for early engagement.

3.*Strengthening organisational responsibility for involvement*

Consistent with research highlighting the structural fragility of PPI ([7]; [12]), the findings suggest that responsibility for involvement is often distributed unevenly and reliant on individual advocacy. Strengthening organisational mechanisms, including clearer roles and authority for PPI leadership, may support more consistent implementation across settings.

4.*Recognition of contributor labour as part of research infrastructure*

While recognition of contributor labour has been widely discussed within the literature, the present findings illustrate how its limited visibility persists across governance processes. This suggests a need to consider how emotional, cognitive and temporal contributions are acknowledged within institutional systems, including through compensation, authorship and formal recognition mechanisms.

5.*Enhancing opportunities for ethical review to engage with involvement*

The findings indicate that Research Ethics Committees often rely on indirect or narrative accounts of involvement when assessing applications. Building on previous work (Staley et al., 2017), this study suggests that creating more structured opportunities for reviewers to examine how experiential knowledge has informed ethical design may enhance the contribution of PPI within governance processes.

Taken together, these implications highlight a need to align institutional processes with the multiple purposes of involvement, including improving research quality, relevance and ethical robustness, while recognising the contextual and relational nature of experiential knowledge.

### Implications for practice

For practitioners working in clinical research systems, the findings highlight several considerations. First, PPI may be more usefully understood as an infrastructural component of research design rather than an optional enhancement. Embedding involvement early in research development may reduce the likelihood of performative engagement and increase the visibility of contributor influence.

Second, teams may benefit from documenting PPI decisions as they occur. This documentation should balance accountability with reflective learning, allowing researchers and contributors to describe both substantive changes and instances where involvement confirmed existing design decisions.

Third, organisations should recognise that sustained PPI cannot rely indefinitely on personal goodwill. Without structural protection, engagement remains vulnerable to workload pressure, funding cycles and leadership change.

Finally, ethics reviewers and governance professionals may benefit from clearer opportunities to interrogate the influence of involvement. Encouraging richer narrative explanations may enhance understanding of how ethical acceptability has been shaped through experiential insight rather than relying solely on procedural indicators.

These implications suggest that meaningful PPI is less a question of persuasion than of design. The challenge is not convincing stakeholders that involvement matters, it is building systems capable of sustaining and learning from it.

## Conclusion

The DANIELLE study demonstrates that PPI occupies a complex and at times paradoxical position within contemporary clinical research systems. Across stakeholder groups, involvement was described as ethically important, experientially meaningful and widely endorsed in principle, yet unevenly supported and protected by institutional structures. Through cross-cohort triangulation, the study shows that tensions surrounding involvement are not isolated shortcomings of practice but recurring features of research system design. Participants did not question the value of PPI itself; rather, they questioned the durability, clarity and consistency of the organisational frameworks intended to sustain it.

By integrating perspectives from contributors, sponsors, professional leads and ethics reviewers, the study highlights that involvement serves multiple purposes within research systems, including improving relevance, feasibility and ethical acceptability through the contribution of experiential knowledge. The findings suggest that meaningful involvement cannot rely indefinitely on personal goodwill, advocacy or informal commitment. Systems that depend primarily on motivation without providing appropriate structural support remain vulnerable to inconsistency and erosion.

As a co-produced inquiry initiated by PPI contributors themselves, the DANIELLE study foregrounds the lived consequences of this fragility. It also demonstrates how involvement functions as a form of knowledge exchange that can strengthen research design when supported effectively. The study therefore suggests that the future of involvement lies not only in reaffirming its ethical importance, but in developing institutional practices that recognise diverse forms of evidence, enable earlier collaboration and support sustained partnership between stakeholders. Strengthening PPI may therefore require a shift from rhetorical endorsement towards adaptive organisational learning, ensuring that the commitments embedded in research culture are matched by systems capable of supporting meaningful and context-sensitive involvement.

## Supplementary Information

Below is the link to the electronic supplementary material.

Supplementary Material 1

## Data Availability

The datasets generated and analysed during the current study are not publicly available due to the sensitive nature of qualitative interview data and the need to protect participant confidentiality. Anonymised excerpts supporting the findings are included within the manuscript. Further information about the dataset may be available from the corresponding author on reasonable request and subject to ethical approval.
