# Supplementary material for: Triangulating patient and public involvement in clinical research: a cross-cohort qualitative study
Source: Res Involv Engagem. 2026 Jun 3;12:80. doi: 10.1186/s40900-026-00918-0 (PMC13231631; doi:10.1186/s40900-026-00918-0)
Supplement: Supplementary file 1 — Supplementary Material 1 [file 40900_2026_918_MOESM1_ESM.docx]

**Appendix: Interview Guides**

*Semi-structured interview topic guides*

***Patient and Public Involvement contributors***

1. Could you tell me how you became involved in Patient and Public Involvement (PPI)?
2. What does being a member of a PPI group involve in practice?
3. What does your involvement mean to you personally?
4. In your view, how important is PPI within clinical research?
5. Can you describe your experiences of providing feedback to researchers?
6. Are you aware of the PPI section within the Integrated Research Application System (IRAS), which is used for permissions and approvals in UK health and social care research? (If not, a brief overview was provided.)
7. What are your views on how PPI is described, evidenced and reported within the IRAS application process?
8. Do you think there could be more effective ways of evidencing or reporting PPI feedback within research governance systems?
9. Are there any additional experiences of being involved in PPI that you would like to discuss?

***Clinical trial sponsor staff***

1. Could you describe your understanding of Patient and Public Involvement in research?
2. In your current role, do you have any involvement with PPI activities or processes?
3. In your view, how important is PPI to clinical trial sponsors, and why?
4. What benefits do you think PPI can bring to clinical research from a sponsor perspective?
5. Do you think the integration of PPI within your organisation or service could be improved?
6. If so, what impact might improved involvement have on research delivery or governance?
7. Is there anything else you would like to discuss regarding PPI in clinical research?

***Professional PPI leads***

1. Could you describe your role and responsibilities as a PPI lead?
2. How do you see the importance of PPI within clinical research?
3. What do you consider to be the main benefits of effective involvement?
4. What are your views on how PPI is currently embedded and implemented within NHS research settings?
5. Are there any changes you believe could strengthen how PPI is integrated into research systems?
6. Is there anything else you would like to discuss regarding PPI in clinical research?

***Research Ethics Committee members***

1. Could you describe your understanding of Patient and Public Involvement in research?
2. From your perspective, do researchers involve PPI sufficiently during the design and development of their studies?
3. How important do you think PPI is within the work of Research Ethics Committees, and why?
4. What benefits do you think PPI can bring to ethical review processes?
5. What are your views on the information provided about PPI within the IRAS application system?
6. How effectively do you think PPI influence is evidenced or communicated within research applications?
7. Is there anything else you would like to discuss regarding PPI within clinical research?
